# Supplementary material for: Cost-effectiveness of livelihood interventions for families of children with cerebral palsy in rural Bangladesh
Source: PLoS One. 2025 Aug 21;20(8):e0326653. doi: 10.1371/journal.pone.0326653 (PMC12370056; doi:10.1371/journal.pone.0326653)
Supplement: S1 File — S1 Table. Sensitivity analysis of lamb. S2 Table. Sensitivity analysis of sewing machine. S3 Table. Sensitivity analysis of goat. S4 Table. Sensitivity analysis of ghee making utensils. (DOCX) [file pone.0326653.s001.docx]

## Supplementary Table 1: Sensitivity analysis of lamb

| Lamb | BDT 3000 | In USD 35.4 | BDT 5000 | In USD 58.9 | BDT 7000 | In USD 82.5 | BDT 9000 | In USD 106.1 | BDT 11000 | In USD 129.7 |
| --- | --- | --- | --- | --- | --- | --- | --- | --- | --- | --- |
| 7 | 875.0 | 10.3 | 14875.0 | 175.4 | 28875.0 | 340.5 | 42875.0 | 505.5 | 56875.0 | 670.6 |
| 9 | 1125.0 | 13.3 | 19125.0 | 225.5 | 37125.0 | 437.7 | 55125.0 | 649.9 | 73125.0 | 862.2 |
| 11 | 1375.0 | 16.2 | 23375.0 | 275.6 | 45375.0 | 535.0 | 67375.0 | 794.4 | 89375.0 | 1053.8 |
| 13 | 1625.0 | 19.2 | 27625.0 | 325.7 | 53625.0 | 632.3 | 79625.0 | 938.7 | 105625.0 | 1245.4 |
| 14 | 1750.0 | 20.6 | 29750.0 | 350.8 | 57750.0 | 680.9 | 85750.0 | 1011.0 | 113750.0 | 1341.2 |
| 15 | 1875 | 22.1 | 31875.0 | 375.8 | 61875.0 | 729.6 | 91875.0 | 1083.3 | 121875.0 | 1437.0 |

Note: No labour cost is included in the calculation because subsistence farming/practices

## Supplementary Table 2: Sensitivity analysis of sewing machine

| Sewing machine | BDT 5000^1^ | In USD 59.0 | BDT 10000 | In USD | BDT 15000 | In USD | BDT 20000 | In USD | BDT 25000 | In USD |
| --- | --- | --- | --- | --- | --- | --- | --- | --- | --- | --- |
| 5 | -3000.0 | -35.4 | 22000.0 | 259.4 | 47000.0 | 554.2 | 72000.0 | 849.0 | 97000.0 | 1143.7 |
| 7 | -4200.0 | -49.5 | 30800.0 | 363.2 | 65800.0 | 775.8 | 100800.0 | 1188.5 | 135800.0 | 1601.2 |
| 9 | -5400.0 | -63.7 | 39600.0 | 467.0 | 84600.0 | 997.5 | 129600.0 | 1528.1 | 174600.0 | 2058.7 |
| 11 | -6600.0 | -77.8 | 48400.0 | 570.7 | 103400.0 | 1219.2 | 158400.0 | 1867.7 | 213400.0 | 2516.2 |
| 13 | -7800.0 | -92.0 | 57200.0 | 674.5 | 122200.0 | 1440.9 | 187200.0 | 2207.2 | 252200.0 | 2973.7 |
| 15 | -9000.0 | -106.1 | 66000.0 | 778.2 | 141000.0 | 1662.5 | 216000.0 | 2546.9 | 291000.0 | 3431.2 |

Note: ^1^Negative figures mean loss.

## Supplementary Table 3: Sensitivity analysis of goat

| Goat | BDT 5000 | In USD 59.0 | BDT 7000 | In USD 82.5 | BDT 9000 | In USD 106.1 | BDT 11000 | In USD 129.7 | BDT13000 | In USD 153.9 |
| --- | --- | --- | --- | --- | --- | --- | --- | --- | --- | --- |
| 70 | 151550.0 | 1786.9 | 291550.0 | 3437.7 | 428750.0 | 5055.4 | 568750.0 | 6706.2 | 708750.0 | 8356.9 |
| 80 | 173200.0 | 2042.2 | 333200.0 | 3928.8 | 490000.0 | 5777.6 | 650000.0 | 7664.2 | 810000.0 | 9550.8 |
| 90 | 194850.0 | 2297.5 | 374850.0 | 4419.9 | 551250.0 | 6499.8 | 731250.0 | 8622.2 | 911250.0 | 10744.6 |
| 100 | 216500.0 | 2552.8 | 416500.0 | 4910.9 | 612500.0 | 7222.0 | 812500.0 | 9580.2 | 1012500.0 | 11938.5 |
| 110 | 238150.0 | 2808.0 | 458150.0 | 5402.0 | 673750.0 | 7944.2 | 893750.0 | 10538.3 | 1113750.0 | 13132.3 |
| 120 | 259800.0 | 3063.3 | 499800.0 | 5893.2 | 735000.0 | 8666.4 | 975000.0 | 11496.3 | 1215000.0 | 14326.1 |

## Supplementary Table 4: Sensitivity analysis of ghee making utensils

| Ghee making utensils | BDT 3000 | In USD 35.4 | BDT 5000^2^ | In USD 59.0 | BDT 7000 | In USD 82.5 | BDT 9000 | In USD 106.1 | BDT 10000 | In USD 117.9 |
| --- | --- | --- | --- | --- | --- | --- | --- | --- | --- | --- |
| 1 | -2000.0 | -23.6 | 0.0 | 0.0 | 2000.0 | 23.6 | 4000.0 | 47.1 | 5000.0 | 59.0 |
| 2 | -4000.0 | -47.2 | 0.0 | 0.0 | 4000.0 | 47.2 | 8000.0 | 94.3 | 10000.0 | 117.9 |
| 3 | -6000.0 | -70.7 | 00 | 0.0 | 6000.0 | 70.7 | 12000.0 | 141.5 | 15000.0 | 176.9 |
| 4 | -8000.0 | -94.3 | 0.0 | 0.0 | 8000.0 | 94.3 | 16000.0 | 188.7 | 20000.0 | 235.8 |
| 5 | -10000.0 | -117.9 | 0.0 | 0.0 | 10000.0 | 117.9 | 20000.0 | 235.8 | 25000.0 | 294.8 |
| 6 | -12000.0 | -141.5 | 0.0 | 0.0 | 12000.0 | 141.5 | 24000.0 | 283.0 | 30000.0 | 353.7 |

Note: ^2^0 means no loss and no profit.
