# Supplementary material for: Cost-effectiveness of livelihood interventions for families of children with cerebral palsy in rural Bangladesh
Source: PLoS One. 2025 Aug 21;20(8):e0326653. doi: 10.1371/journal.pone.0326653 (PMC12370056; doi:10.1371/journal.pone.0326653)
Supplement: Attachment — Submitted filename: Authors responses to editors comments.docx [file pone.0326653.s003.docx]

**Authors’ responses to reviewers’ comments**

**Title:** Cost-effectiveness of livelihood interventions for families of children with cerebral palsy in rural Bangladesh

| **Editor’s comments** | **Authors’ response** |
| --- | --- |
| When submitting your revision, we need you to address these additional requirements.  1. Please ensure that your manuscript meets PLOS ONE's style requirements, including those for file naming. The PLOS ONE style templates can be found at [https://journals.plos.org/plosone/s/file?id=wjVg/PLOSOne_formatting_sample_main_body.pdf](https://url.au.m.mimecastprotect.com/s/KTEHCWLV1DS5v5pqpHpujuoqPbv?domain=journals.plos.org) and  [https://journals.plos.org/plosone/s/file?id=ba62/PLOSOne_formatting_sample_title_authors_affiliations.pdf](https://url.au.m.mimecastprotect.com/s/TS6cCXLWPESX3X6K6u7CguWvUZ_?domain=journals.plos.org) | We have now revised the manuscript and fixed the all formatting issues as per the guideline. |
| 2. Thank you for stating the following financial disclosure: This trial was funded by the Research Foundation of Cerebral Palsy Alliance (PG02218—Support CP Trial) and international funding from CSF Global Bangladesh and awarded to Prof Gulam Khandaker.    Please state what role the funders took in the study.  If the funders had no role, please state: ""The funders had no role in study design, data collection and analysis, decision to publish, or preparation of the manuscript.""  If this statement is not correct you must amend it as needed.  Please include this amended Role of Funder statement in your cover letter; we will change the online submission form on your behalf. | We have now revised the financial statement as follows as per you comment-  **Funding**  *This trial was funded by the Research Foundation of Cerebral Palsy Alliance (PG02218—Support CP Trial) and international funding from CSF Global Bangladesh and awarded to Prof Gulam Khandaker. The funders had no role in study design, data collection and analysis, decision to publish, or preparation of the manuscript.*  We have particularly mentioned it to the cover letter with the request to update as per suggestion of the journal office. |
| 3. Thank you for stating the following in the Acknowledgments Section of your manuscript: We extend sincere gratitude to primary caregivers and children with CP for their valuable time and voluntary involvement in the SUPPORT CP trial. Additionally, we express heartfelt appreciation to the dedicated CSF global team in Bangladesh for their diligent efforts and support. This trial was funded by the Research Foundation of Cerebral Palsy Alliance (PG02218—Support CP Trial) and international funding from CSF Global Bangladesh. The authors declare that there is no conflict of interest. Research data can be accessed by contacting the corresponding author.  We note that you have provided funding information that is not currently declared in your Funding Statement. However, funding information should not appear in the Acknowledgments section or other areas of your manuscript. We will only publish funding information present in the Funding Statement section of the online submission form.  Please remove any funding-related text from the manuscript and let us know how you would like to update your Funding Statement. Currently, your Funding Statement reads as follows: This trial was funded by the Research Foundation of Cerebral Palsy Alliance (PG02218—Support CP Trial) and international funding from CSF Global Bangladesh and awarded to Prof Gulam Khandaker.  Please include your amended statements within your cover letter; we will change the online submission form on your behalf. | We have now revised the acknowledgement section as per your comment. We have removed sentences relevant for funding and conflict of interest and presented them in different relevant sections. The revised acknowledgement section now reads-  **Acknowledgment**  *We extend sincere gratitude to primary caregivers and children with CP for their valuable time and voluntary involvement in the SUPPORT CP trial. Additionally, we express heartfelt appreciation to the dedicated CSF global team in Bangladesh for their diligent efforts and support.* |
| 4. We notice that your supplementary tables are included in the manuscript file. Please remove them and upload them with the file type 'Supporting Information'. Please ensure that each Supporting Information file has a legend listed in the manuscript after the references list. | We have removed all supplementary tables from the manuscript and presented them in separate file named “Supporting information”. |
| 5. We note you have included a table to which you do not refer in the text of your manuscript. Please ensure that you refer to Table 1 in your text; if accepted, production will need this reference to link the reader to the Table. | Many thanks for bringing this to our attention. We have now ensured all tables are properly cited. |
| 6. Please review your reference list to ensure that it is complete and correct. If you have cited papers that have been retracted, please include the rationale for doing so in the manuscript text, or remove these references and replace them with relevant current references. Any changes to the reference list should be mentioned in the rebuttal letter that accompanies your revised manuscript. If you need to cite a retracted article, indicate the article’s retracted status in the References list and also include a citation and full reference for the retraction notice. | We have checked all references and ensured they all are properly cited. Please have a look. |
