# Supplementary material for: Cost-effectiveness of livelihood interventions for families of children with cerebral palsy in rural Bangladesh
Source: PLoS One. 2025 Aug 21;20(8):e0326653. doi: 10.1371/journal.pone.0326653 (PMC12370056; doi:10.1371/journal.pone.0326653)
Supplement: Attachment — Submitted filename: Authors responses to reviewers comments.docx [file pone.0326653.s004.docx]

| **Comments of Reviewer # 1** | **Authors’ response** |
| --- | --- |
| This is a very interesting study about the children with cerebral palsy. However, there are following issue that need further classification-   1. The severity of CP patients, whether they can live independently, and the basic expenses for treatment and rehabilitation. 2. How many family members were needed to take care of in their daily lives? 3. What are the urgent problems that these patients and families need to solve? | We have now added the following sentences to address your comment-  *……..* *Neurodevelopmental disorders are the leading cause of childhood disability globally, with cerebral palsy (CP) being the most common among them [1, 2]. This is further higher in low- and middle-income countries (LMICs) such as Bangladesh, as like other form of disability, compared to high-income countries [3, 4]. In Bangladesh, the population-based prevalence of CP has been estimated to be 3.4 per 1000 children [5]. A majority of these children (68.2%) experience moderate to severe gross motor impairments, which hinder their ability to perform everyday activities [6]. As such, these individuals predominantly rely on their families to sustain their basic needs. In most cases, at least one dedicated caregiver- typically the mother, supported by other family members- is required to assist with day-to-day functioning, as social support from both governmental and non-governmental sectors remains insufficient [7, 8]. One of the most pressing problems is the limited access to rehabilitation services for children with CP in Bangladesh. When such services are available, they are often prohibitively expensive, making them inaccessible to most families due to widespread economic constraints [9]. As a result, families urgently need affordable, accessible rehabilitation services and stronger institutional support to manage the long-term care and well-being of children with CP. However, this support is lacking in LMICs, including Bangladesh.*  *Such dependency of children with CP on their caregivers for day-to-day activities places the caregivers in disadvantaged positions, including a significant reduction in their workforce participation compared to the general population [10]. This has a considerable impact on their economic stability, directly affecting the caregivers' well-being and quality of life [11], and indirectly influencing the well-being of children with CP by limiting the caregivers’ income, which could otherwise support their increasing healthcare needs [12, 13]. Addressing these challenges is therefore a priority to ensure the well-being of children with CP, their caregivers, and their extended families. ………* |
| **Comments of Reviewer#2** |  |
| In lines 76-80, it is recommended that the authors first provide a comprehensive introduction to the clinical characteristics of cerebral palsy, followed by an in-depth discussion on how this condition exacerbates the socioeconomic burden on families from low-income grounds. | We have rewritten the first two paragraphs of the introduction section as follows to address your comment as well as the first reviewer’s comment-  *Neurodevelopmental disorders are the leading cause of childhood disability globally, with cerebral palsy (CP) being the most common among them. This is further higher in low- and middle-income countries (LMICs) such as Bangladesh, as like other prevalence of other form of disability, compared to high-income countries [1, 2]. In Bangladesh, the population-based prevalence of CP has been estimated to be 3.4 per 1000 children [3]. A majority of these children (68.2%) experience moderate to severe gross motor impairments, which hinder their ability to perform everyday activities [4].*  *These individuals predominantly rely on their families to sustain their basic needs. In most cases, at least one dedicated caregiver- typically the mother, supported by other family members- is required to assist with day-to-day functioning, as social support from both governmental and non-governmental sectors remains insufficient [5] [6]. One of the most pressing problems is the limited access to rehabilitation services for children with cerebral palsy in Bangladesh. When such services are available, they are often prohibitively expensive, making them inaccessible to most families due to widespread economic constraints [7]. As a result, families urgently need affordable, accessible rehabilitation services and stronger institutional support to manage the long-term care and well-being of children with CP. However, this support is lacking in LMICs, including Bangladesh, where these groups often depend on caregivers for their day-to-day needs, placing them in a disadvantaged position.*  *“Neurodevelopmental disorders are the leading cause of childhood disability globally, with cerebral palsy (CP) being the most common among them. Recent estimates indicate that the prevalence of childhood disability is substantially higher in low- and middle-income countries (LMICs) such as Bangladesh compared to high-income countries [1, 2]. In Bangladesh, the population-based prevalence of CP has been estimated to be 3.4 per 1000 children [3]. A majority of these children (68.2%) experience moderate to severe gross motor impairments, which hinder their ability to perform everyday activities [4].*  *These individuals predominantly rely on their families to sustain their basic needs. In most cases, at least one dedicated caregiver- typically the mother, supported by other family members- is required to assist with day-to-day functioning, as social support from both governmental and non-governmental sectors remains insufficient [5] [6]. In Bangladesh, access to rehabilitation for children with CP is very limited and when available the cost is often prohibitively high due to economic constrains [7]……………* |
| More detailed inclusion and exclusion criteria for the study samples should be added. | We have now included the following sentences in the study design and setting section of the methods to address your comment-  *……..The following inclusion criteria were considered for participant selection: (1) being the primary caregiver of a child with CP aged five years or younger, and (2) belonging to a family categorised as ultra-poor based on the World Bank criterion of less than US$1.90 per day per capita income [17]. Please note that in September 2022, the World Bank revised the poverty threshold for defining the ultra-poor to US$2.15 per day. However, since our data collection was completed prior to the implementation of this updated threshold, we have used the earlier cut-off value in our analysis…* |
| In lines 143-144, “After distributing the livelihood assets, relevant training sessions were organised to develop participants skills and knowledge.” Please provide more detailed information about these training sessions, such as whether the proficiency levels of each participant are comparable. | We have written the following sentences in the livelihood support section of the methods to address your comment -  …..*After asset distribution, a veterinary surgeon from the local government livestock department provided a three-day hands-on training for participants on scientific livestock rearing practices. This training covered construction of animal shelters, preparation of balanced diets, identification and management of common diseases, and basic veterinary care. In parallel, other local experts delivered relevant training tailored to non-livestock livelihood activities, enhancing participants’ skills and knowledge in income-generating activities. Pre- and post-training evaluations were conducted to identify specific knowledge gaps. Follow-up training sessions were organized to address these gaps, ensuring participants could manage their assets effectively to generate income*……. |
| Please supplement the methodology section with the methodology for sensitivity analysis. | Many thanks for your suggestions. We agree that including a sensitivity analysis could help address some unexamined aspects of our study. However, we were unable to incorporate this into the manuscript due to the lack of quantitative data. The number of recipients for each type of support was too small to allow for meaningful quantitative analysis. For example, only three participants received chickens, and only one received ghee-making tools. Given these limitations, conducting a sensitivity analysis was not feasible.  Nevertheless, we have now acknowledged this limitation in the manuscript by adding the following sentences to the Strengths and Limitations section*..In addition, participants’ background characteristics—such as education, occupation, and other factors that could influence the effective use of received investments and the potential for better returns—were important considerations. However, we were unable to explore these factors quantitatively due to the lack of disaggregated data across the different types of livelihood support received. Nonetheless, these aspects were examined as thoroughly as possible through qualitative exploration…*  We have also revised Table 2 to include the number of beneficiaries by type of support received, to help readers better understand the challenges associated with quantitative analysis. |
| How the total return obtained by participants during the investment period determined, and how reliable is it? The authors need to offer more comprehensive methodological explanation to enhance reader’s confidence in the accuracy of the results. | Thank you for this important comment. To determine the total return obtained by participants during the investment period, financial statements were collected through structured interviews conducted during bi-monthly home visits by trained field coordinators. These statements included detailed information on income and expenditures related to the livelihood assets provided.  To enhance reliability, the reported figures were cross verified against prevailing local market prices and validated through triangulation with physical asset checks and observations of production outputs (e.g., number of eggs, litres of ghee, or livestock growth). Any discrepancies were resolved through follow-up visits and consultation with the participants.  Outcomes were independently assessed by a blinded assessor at three time points: baseline, 6 months, and 12 months. The total return was calculated as the cumulative net income generated over the 12-month period, based on participant-reported financial statements that had been verified for accuracy. This approach ensured both the methodological rigor and credibility of the financial outcomes presented in the study. |
| Does the educational level and occupation of the parents in different families indirectly affect the rate of return on investment? | Please see our response to your earlier comment.  We appreciate your thoughtful feedback and fully acknowledge the importance of your suggestion. However, we were unable to implement it due to the lack of disaggregated data by type of livelihood support received. |
| 1. The credibility of results obtained solely based on differences in the rate of return on investment is relatively low, it is necessary to employ more statistical methods, such as linear regression, to determine whether there is significance. | We appreciate your thoughtful comment and fully understand the value of your suggestion. Although we had initially planned to explore this, we were ultimately unable to proceed due to two main reasons:   1. Comparing returns across different types of support raises statistical concerns, as the maximum potential return inherently varies by support type—even under ideal conditions. This would challenge the validity of direct comparisons. 2. The sample sizes for the various livelihood support categories were too small to be included in a regression model, which would lead to highly unstable and unreliable estimates. For example, only three participants received chickens, and just one received ghee-making tools. |
| 2. Conducting subgroup analyses based on participants backgrounds is indeed necessary. | Please see our response to your earlier comments. |
| 3. The line numbers disappeared after the tenth page. | The line numbers have been corrected. |
| 4. In the section, “Lesson learned about different livelihood activities and the key to success,” the conclusions are partially subjective and should be supported by an appropriate number of references. | Relevant references are added in this portion. |
| 5. In the discussion section, “To the best of our knowledge, this study compares the efficacy of various livelihood support interventions to determine the most effective approach, is a pioneering effort within LMIC settings.” Please explicitly state that this is the most effective approach for improving the well-being of families with children affected by cerebral palsy. | We have revised the relevant statement follows to address your comment-  *To the best of our knowledge, this is the first study within LMIC settings to compare the efficacy of various livelihood support interventions in order to identify the most effective approach for improving the wellbeing of families with children affected by CP.* |
| The study primarily focuses on the livelihoods of families with children affected by cerebral palsy. However, in the discussion section, there is insufficient analysis and discussion regarding this specific population group. | We have revised the discussion section and added sentences to more broadly address the challenges faced by children with cerebral palsy (CP) and their caregivers. The relevant paragraphs with the newly added content now read as follows:  *Families of children with CP in rural Bangladesh face unique challenges due to the combined burden of poverty and disability. In this study, 91.3% of the primary caregivers were mothers, most of whom were homemakers with limited education—only 38.8% completed primary education, and 7.5% had no formal education. The vast majority of families (76.2%) had only one income earner, typically engaged in low-paid agricultural or blue-collar work (81%). Over half of the households had four or more members, intensifying financial strain. These circumstances severely limited caregivers’ ability to prioritize rehabilitation for their children. Livelihood interventions tailored to such contexts—such as small livestock or home-based enterprises—offered practical, income-generating opportunities that aligned with caregivers’ existing responsibilities. This study highlights the importance of disability-inclusive livelihood programs as a critical strategy to improve the well-being of families with children with CP in low-resource settings.* |
